# Characterization of small intestinal neuroendocrine tumorlets

**DOI:** 10.1530/ERC-25-0425

**Published:** 2026-04-13

**Authors:** Akitada Yogo, Naoki Akanuma, Grace E Kim, Alan Paciorek, Li Zhang, Phi Le, Kenzo Hirose, Carlos U Corvera, Emily K Bergsland, Eric K Nakakura

**Affiliations:** ^1^ Division of Surgical Oncology, Section of Hepatopancreaticobiliary Surgery, Department of Surgery, University of California, San Francisco, California, USA; ^2^ Helen Diller Family Comprehensive Cancer Center, University of California, San Francisco, California, USA; ^3^ Department of Pathology and Laboratory Medicine, University of California Davis, Sacramento, California, USA; ^4^ Department of Pathology, University of California, San Francisco, California, USA; ^5^ Department of Epidemiology and Biostatistics, University of California, San Francisco, California, USA; ^6^ Division of Hematology and Oncology, Department of Medicine, University of California, San Francisco, California, USA

**Keywords:** neuroendocrine tumors, small intestine, neoplasm micrometastasis, clonal evolution

## Abstract

Small intestine neuroendocrine tumors (SI-NETs) frequently present as multifocal primaries. We commonly observe microscopic lesions in the superficial layer of the small intestine of SI-NET patients. We aimed to define them as small intestinal neuroendocrine tumorlets (SINTs) and explore their clinical and biological significance. Twenty multifocal and twenty unifocal SI-NETs patients who received resection at a single institution were retrospectively reviewed. Four hundred and forty six archived pathological slides were examined for microscopic lesions located in the lamina propria, muscularis mucosa, and superficial submucosa. Clinicopathological associations and progression-free survival were analyzed. Previously published genomic data were re-analyzed. SINTs were identified in 50% of multifocal and 30% of unifocal SI-NET patients. Median SINT size was 95 μm, with a median distance of 2.2 mm from the nearest mass. Compared to the ‘true unifocal’ group (unifocal without SINT), the ‘multifocal-spectrum’ group (multifocal or unifocal with SINT) had higher BMI (median: 27.6 vs 22.8, *P* = 0.0060), higher rates of perineural invasion (OR: 5.5, *P* = 0.044), larger mesenteric mass (median: 2.6 vs 1.6 cm, *P* = 0.034), and more advanced pT stage (pT3 or pT4, OR: 7.2, *P* = 0.018). Genomic re-analysis suggested that 13% of cells in multifocal primary tumors could share clonal origins, possibly indicating clonal spread via SINTs. SINTs may serve as a new biomarker for multifocal spectrum with local aggressiveness. The actual frequency of multifocal SI-NET may be higher than currently recognized in clinical practice. Further studies are needed to validate their prognostic and biological significance.

## Introduction

Small intestine neuroendocrine tumors (SI-NETs) have an increasing incidence and account for 37% of all small intestinal malignancies in the United States, making them the most common small intestinal neoplasm (1, 2). They are characterized by a high frequency of multifocal primary tumors (45–54%), and surgeons intraoperatively palpate the entire jejunum–ileum to identify all tumors to ensure complete resection because each primary has the capacity to metastasize (3, 4, 5, 6, 7). However, in surgically resected specimens from multifocal SI-NET patients, we often identify additional non-palpable, microscopic tumor foci in the superficial layers of the small intestine. Notably, such microscopic lesions in SI-NETs have not been investigated (4, 8, 9, 10).

Tumor micronodules have been described in several organs, often representing either precursor or satellite lesions. Pulmonary carcinoid tumorlets (PCTs), small proliferations of neuroendocrine cells (≤0.5 cm) that invade the basement membrane, are often associated with chronic inflammation and diffuse idiopathic pulmonary neuroendocrine cell hyperplasia (DIPNECH) and are considered the possible origin of pulmonary carcinoid tumors (11, 12). In the stomach, nodular enterochromaffin-like cell proliferation is observed with autoimmune gastritis and is considered neoplastic when the expansile nodule reaches a size of 0.5 mm (13, 14). Pancreatic neuroendocrine microadenomas (≤0.5 cm) in patients with MEN1 or VHL mutation are believed to be precursor lesions for pancreatic NET with high metastatic potential (15, 16, 17). In contrast, microscopic tumor buds in colorectal adenocarcinoma, as well as microsatellites and extratumoral intralymphatic or perineural tumor foci in melanoma and oral cavity squamous cell carcinoma, are markers of local dissemination and poor prognosis (18, 19, 20, 21).

In SI-NETs, microscopic perineural/lymphovascular invasion mainly in the deeper layers of the small intestine (e.g. submucosa, muscularis propria, and subserosa/serosa) is recognized to be associated with higher metastatic and lower complete resection rates (22). However, non-palpable microscopic lesions in the superficial layers, particularly those separate from palpable masses, remain uninvestigated, and their biological and clinical significance is unclear.

We hypothesize that these microscopic lesions may represent early proliferative or disseminated tumor foci contributing to multifocal tumorigenesis in SI-NETs. This study aimed to characterize these lesions, which we propose as small intestinal neuroendocrine tumorlets (SINTs), and to assess their clinicopathological significance and potential biological roles.

## Materials and methods

### Study design, patients, and IRB

This study utilized an exploratory, descriptive, and comparative cross-sectional design with survival analysis based on a single-institution retrospective cohort. A stratified sampling approach was employed to select 40 patients who underwent surgical resection of primary SI-NETs at UCSF between March 2019 and November 2022. Twenty patients with multifocal SI-NETs and 20 with unifocal SI-NETs were deliberately selected from our institutional database. Inclusion criteria were i) histologically confirmed small intestinal neuroendocrine tumor, ii) surgical resection of primary tumors, iii) availability of sufficient pathological specimens for review, and iv) sufficient clinical and pathological data. Exclusion criteria included i) insufficient pathological specimens for review and ii) incomplete clinical follow-up data. The study was conducted in accordance with the Declaration of Helsinki and HIPAA regulations and was approved by the UCSF Institutional Review Board. Informed consent was waived because of the retrospective design and the use of anonymous patient data.

### Small intestinal neuroendocrine tumorlet (SINT)

From the resected specimens, archived pathological slides were reviewed that included primary tumors, adjacent normal intestinal tissues, uninvolved intestinal tissues, and surgical margins for the detection of subclinical microscopic lesions in the superficial layers of the small intestine. Initial screening was performed by surgeons (AY and EKN), and all candidate lesions were subsequently reviewed by an expert pathologist (GEK), who confirmed tumoral lesions, identified additional tumoral lesions missed during screening, and excluded non-tumoral findings. The superficial layers of the small intestine were defined as the lamina propria, muscularis mucosa, and the superficial submucosa immediately adjacent and deep to the muscularis mucosa, where subclinical microscopic lesions were often found. Subclinical microscopic lesions were defined as surgically non-palpable neuroendocrine tumor lesions with the absence of tumor cell contiguous with an outlined palpable tumor mass. Outlining of the lesions and measurement of diameter and distance were performed on scanned whole-slide images using QuPath (v.0.5.0) software (23). These lesions are termed small intestinal neuroendocrine tumorlet (SINT) hereafter.

### Variables and data sources

Data were collected from medical records, preoperative and postoperative imaging, operative reports, and pathology reports. Patient demographics and some tumor characteristics (size of the largest primary tumor and tumor grade) were considered as potential exposures, whereas other tumor characteristics (i.e., number of primary tumors, perineural invasion, lymphovascular invasion, size of mesenteric mass, pT, pN, M, and stage), surgical outcomes (i.e., positive local surgical margin and postoperative complications), and progression-free survival (PFS) were considered as outcomes. PFS was defined as the number of months from the surgery date to the disease progression or death from any cause. Disease progression was determined based on interpretation by radiologists using radiological imaging. The positive local surgical margin was defined if tumor cells were at the resected proximal or distal intestinal specimen margin. Postoperative complications were graded using the Clavien-Dindo classification.

### Statistical analysis

Continuous variables are represented as medians with interquartile ranges, and categorical variables are represented as frequencies and percentages. The Wilcoxon rank-sum and Fisher’s exact test were used to compare distributions across groups. Survivals were compared using the Kaplan–Meier method and the log-rank test. Statistical analyses and representations were performed using R (v.4.1.1) (Foundation for Statistical Computing, Vienna, Austria) with R packages multcomp (v.1.4–25), survival (v.3.5–5), ggplot2 (v.3.4.2), and survminer (v.0.4.9). Effect sizes were calculated with Cohen’s h (2*asin(sqrt(p1)) – 2*asin(sqrt(p2))) or Cohen’s d (cohen.d() function of effsize (v0.8.1) R package). Post hoc sample size calculation was performed with power.prop.test() function of stats (v4.1.1) R package and ssizeCT.default() function of survSNP (v.0.26) R package with a significance level of *P* < 0.05 and power of 0.8.

### Immunohistochemistry (IHC) of lymphatic vessels

Tissue blocks were sectioned at 5 μm thickness and were stained using podoplanin primary antibody D2–40 (M3619, Agilent/Dako, USA, 1:50). Antigen retrieval was performed for 20 min using BOND Epitope Retrieval Solution 2 (Leica Biosystems, Germany) on the BOND-III Fully Automated IHC and ISH Staining System (Leica Biosystems) to detect lymphatic endothelium.

### Additional genomic subclonal composition analysis

Previous genomic studies reported that multifocal SI-NET tumors had independent clonality and lacked shared driver mutations (24, 25). However, those studies identified a limited number of shared somatic single-nucleotide variants (SNVs) and indels among some of the tumors, suggesting potential shared clonality among multifocal primary tumors. To further investigate their clonal relationships based on cellular subpopulation, we re-analyzed one of the previously published genomic datasets (24). The used dataset was downloaded from an open-access source (Mäkinen *et al.*, Table S2 (see the Supplementary materials given at the end of the article) on 21/1/2025, https://static-content.springer.com/esm/art%3A10.1186%2Fs13073-022-01083-1/MediaObjects/13073_2022_1083_MOESM3_ESM.xlsx) (24). Subclones were inferred from somatic mutation patterns with tumoral variant allele frequency (VAF) in each patient, using LICHeE (v1.0, https://github.com/viq854/lichee) according to the developer’s instructions (26). The probability of observing 12 shared variants between P2 and P3 in patient #876 was calculated using a hypergeometric model with dhyper() function in stats (v.4.1.1) R package with the parameters: *x* = 12, *m* = 1,024, *n* = 3110748599 – 1,024, *k* = 1,484.

## Results

### Descriptive analysis of small intestinal neuroendocrine tumorlets (SINTs)

**Figure 1:**
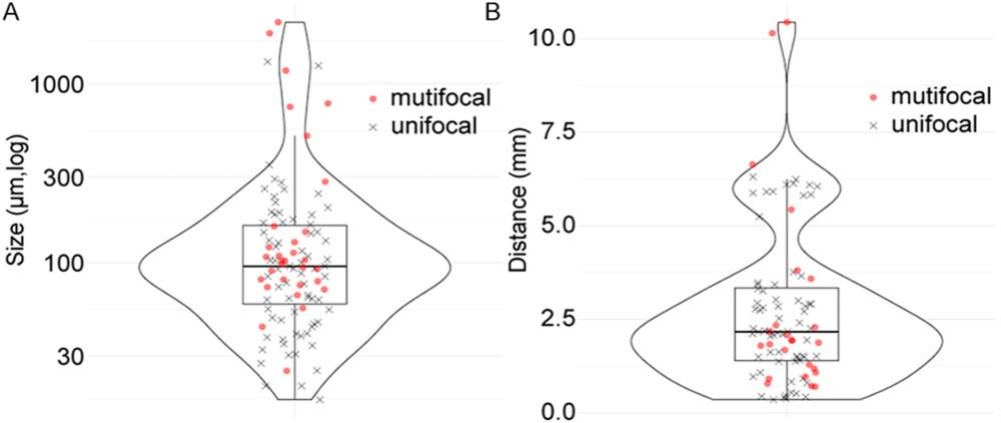
Characteristics of the SINTs. (A) Size of the 103 SINTs. (B) Distance from the nearby palpable SI-NET mass to the 93 SINTs that were detected on the same slide as the palpable SI-NET mass.

**Figure 2:**
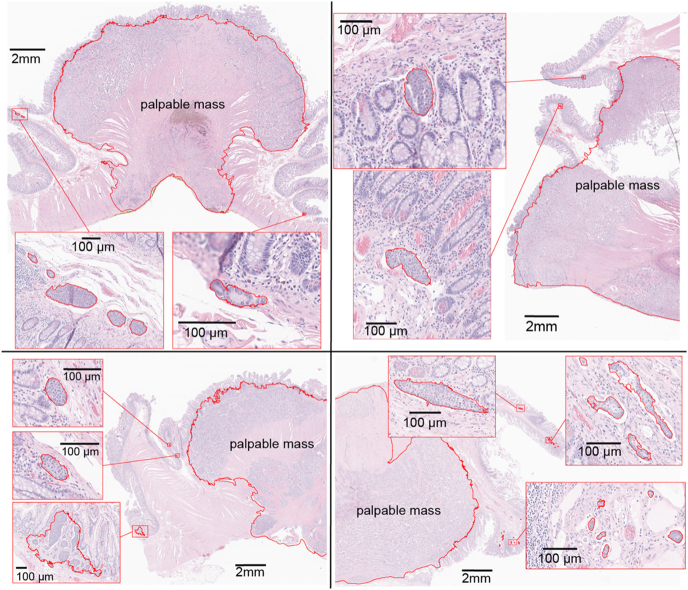
SINTs near palpable mass. Palpable SI-NET masses and SINTs are outlined in red. Hematoxylin and eosin stain.

**Figure 3:**
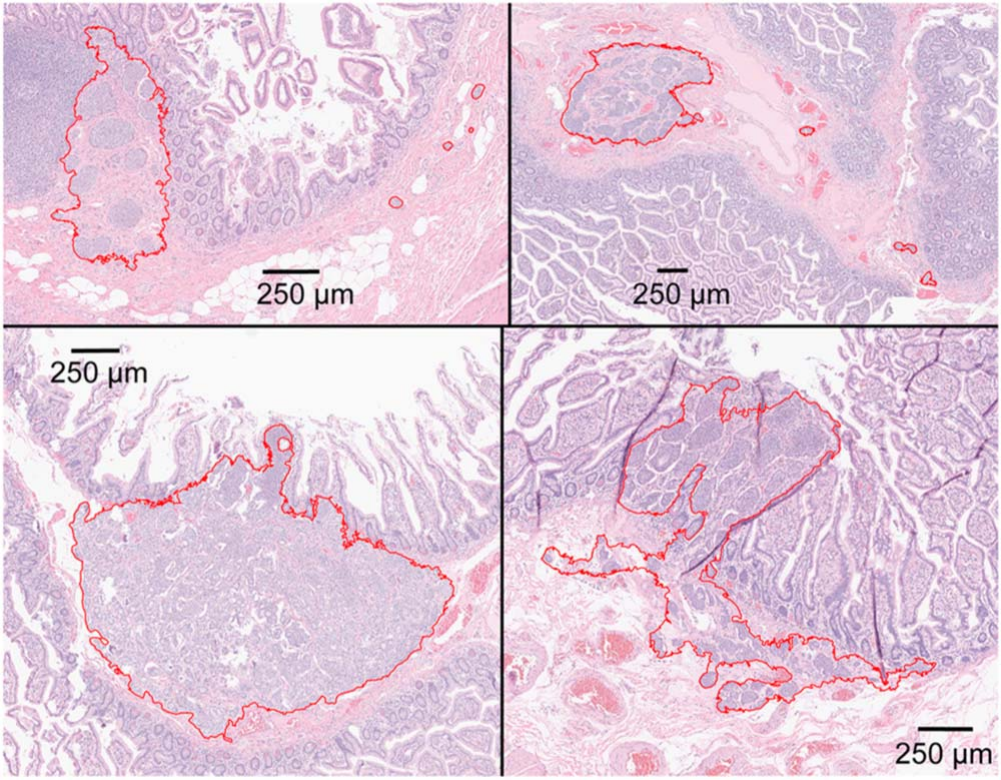
SINTs without adjacent palpable mass. SINTs are outlined in red. Hematoxylin and eosin stain.

**Figure 4:**
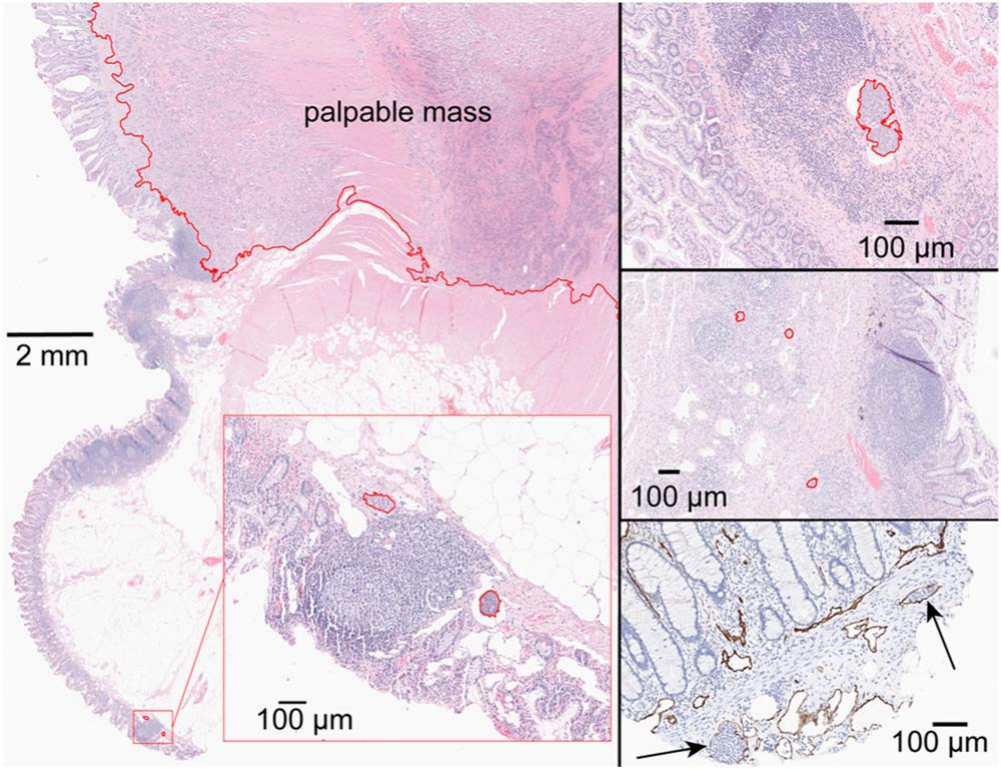
SINTs located within lymphatic vessels. A palpable SI-NET mass and SINTs are outlined in red. Hematoxylin and eosin stain (left, top-right, and middle-right panels). D2–40 immunohistochemistry highlights SINTs within lymphatic vessels (bottom-right panel, arrows).

Pathological review was performed on samples from 40 SI-NET patients (20 multifocal, 20 unifocal, Supplementary Table 1). A total of 446 archived pathological slides were reviewed, comprising 247 slides from multifocal SI-NET cases with 114 primary tumors and 199 slides from unifocal SI-NET cases with 20 primary tumors. SINTs were identified in 10 out of 20 patients with multifocal SI-NET, and in 6 out of 20 patients with unifocal SI-NET. No clear difference in prevalence was observed between the two groups (odds ratio (OR): 2.3 (95% CI: 0.53–10), *P* = 0.33). A median of 4.5 (range: 1–30) SINTs was identified in each patient, with a total of 103 detected in all cases. The median size of the lesions was 95 μm (range: 17–2,200; IQR: 59–160, Fig. 1A). Most of the SINTs, 92 of 103 (89%), were found on the same slide as the palpable SI-NET mass, with a median distance of 2.2 mm from the palpable mass (range: 0.35–1,0; IQR: 1.4–3.3, Figs 1B and 2). The remaining 11 SINT lesions were found in four patients in a section that did not have a palpable mass; five of the SINTs were 0.3 cm from the resection margin in one patient (Fig. 3). The locations in the small intestine were as follows: the lamina propria, *n* = 21 (20%); muscularis mucosa or submucosa, *n* = 79 (77%); and spanning from lamina propria to submucosa, *n* = 3 (3%). Some SINT lesions appeared to be located within lymphatic vessels (Fig. 4).

### Comparative analysis on ‘multifocal-spectrum’ SI-NET

Two of the SINTs in the unifocal SI-NET patients were large enough (1.3 mm each, Fig. 1A) to be considered additional primary tumors. Since no standardized size criteria exist for classifying microscopic lesions as primary tumors, and other SINTs in the unifocal SI-NET patients (17–350 μm) may also develop into new palpable primary tumor masses over time, clinically defined multifocal SI-NETs and unifocal SI-NETs with SINTs of any size may represent a single biological spectrum rather than distinct entities. Accordingly, we re-classified patients into two groups: ‘multifocal-spectrum SI-NET,’ including clinically defined multifocal SI-NET patients and clinically defined unifocal SI-NET patients with SINT, and ‘true unifocal SI-NET,’ including clinically defined unifocal SI-NET patients without SINT. The ‘multifocal-spectrum SI-NET’ patients had a higher BMI (median: 27.6 vs 22.8, *P* = 0.0060, Table 1), higher rate of perineural invasion (PNI) (88 vs 57%, OR: 5.5 (95% CI: 0.91–42), *P* = 0.044), larger size of mesenteric mass (median: 2.6 vs 1.6 cm, *P* = 0.034), and more advanced pT stage (pT3 or pT4, 88 vs 50%, OR: 7.2 (95% CI: 1.2–55), *P* = 0.018), than ‘true unifocal’ SI-NET, but there was no clear difference in the size of the largest primary tumor (median: 2.0 vs 1.7 cm, *P* = 0.34), tumor grade (*P* = 0.66), or frequency of distant metastasis (46 vs 21%, OR: 3.1 (95% CI: 0.6–21), *P* = 0.18).

**Table 1:** Demographics of the cohort by multifocal spectrum.

Characteristic	*n*	‘Multifocal-spectrum,’ *n* (%)		*P*-value
		No, *n* = 14	Yes, *n* = 26	
Age, median (IQR)	40	68 (57, 71)	61 (50, 68)	0.33
Gender female	40	8 (57)	8 (31)	0.18
BMI (kg/m^2^), median (IQR)	40	22.8 (21.9, 26)	27.6 (25.2, 32)	0.0060
Race white	40	12 (86)	17 (65)	0.27
Carcinoid syndrome	40	4 (29)	12 (46)	0.33
Urine 5-HIAA (mg/24 h)	25	6 (5, 126)	14 (7, 40)	0.61
History of small bowel obstruction	40	4 (29)	5 (19)	0.69
Size of the largest primary tumor (cm), median (IQR)	40	1.7 (1.1, 3.0)	2.0 (1.3, 3.0)	0.35
Tumor grade	40			0.66
1		8 (57)	18 (69)	
2		6 (43)	7 (27)	
3		0 (0)	1 (3.8)	
Number of primary tumors, median (IQR)	40	1 (1, 1)	3 (2, 7)	2.3e^−5^
Perineural invasion	40	8 (57)	23 (88)	0.044
Lymphovascular invasion	40	10 (71)	19 (73)	1
Size of mesenteric mass (cm), median (IQR)	40	1.3 (0.0, 3.6)	2.6 (1.5, 3.8)	0.034
pT staging	40			0.018
pT1/pT2		7 (50)	3 (12)	
pT3/pT4		7 (50)	23 (88)	
pN +	40	12 (86)	25 (100)	0.12
M staging	40			0.18
M0		11 (79)	14 (54)	
M1a/1b/1c		3 (21)	12 (46)	
Stage	40			0.068
I		2 (14)	0 (0)	
III		9 (64)	14 (54)	
IV		3 (21)	12 (46)	
Positive local surgical margin	40	0 (0)	1 (3.8)	1
Postoperative complications >= G3	40	0 (0)	0 (0)	NA
Survival status dead	40	0 (0)	2 (7.7)	0.53
Follow-up months, median (IQR)	40	54 (40, 60)	39 (32, 47)	0.076

BMI, body mass index; 5-HIAA, 5-hydroxyindoleacetic acid; NA, not applicable.

**Figure 5:**
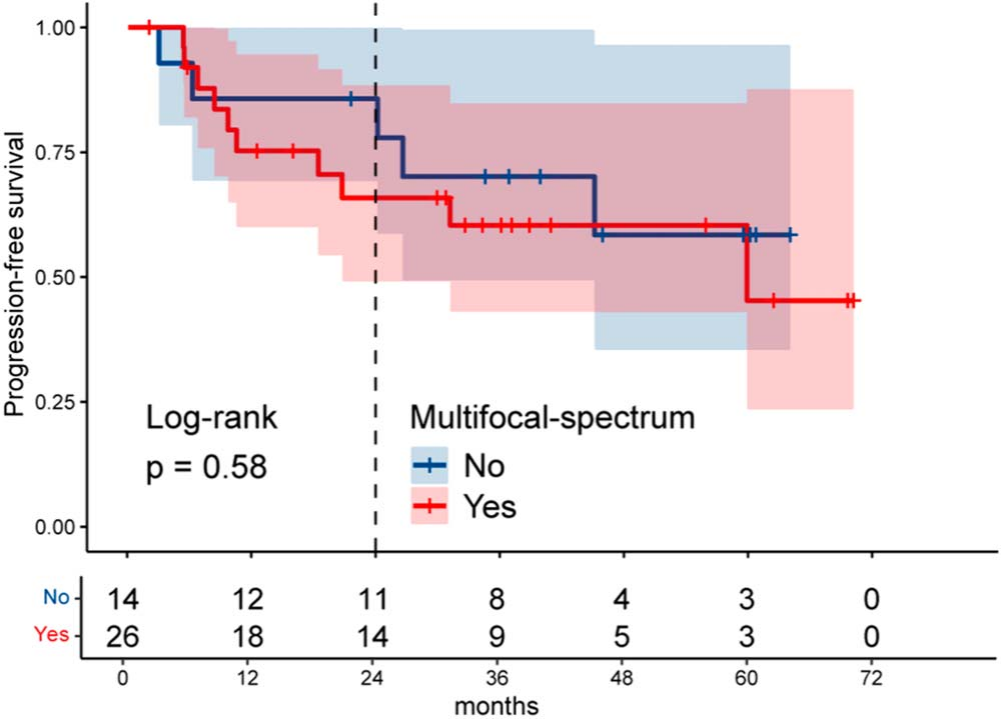
Survival curves by ‘multifocal spectrum.’ Kaplan–Meier progression-free survival curves are shown for the patients with ‘multifocal spectrum’ (red) and ‘true unifocality’ (blue).

There was no clear difference in PFS by ‘multifocal spectrum’ (2-year PFS rates are 66% (95% CI: 49–88%) for ‘multifocal-spectrum’ vs 86% (69–100%) for ‘true unifocal’ patients; hazard ratio: 1.4 (95% CI 0.46–4.0); *P* = 0.58, Fig. 5). Meanwhile, when comparing clinically defined multi- and unifocal patients, all variables were similar except for the medium-sized difference in the size of mesenteric mass (median 3.0 vs 1.6 cm, *P* = 0.045, effect size = 0.65, Supplementary Table 1), including PFS (2-year PFS rates are 78% (95% CI: 61–100%) for multifocal vs 70% (52–93%) for unifocal patients; hazard ratio: 0.63 (95% CI: 0.23–1.8); *P* = 0.40).

### Additional genomic subclonal composition analysis

Based on the hypothesis that SINTs originate from an existing primary tumor and subsequently give rise to new tumor masses, we re-analyzed a previously published genomic dataset to investigate potential clonal relationships underlying multifocal SI-NETs (24).

The dataset included 11 patients with multifocal SI-NET, comprising a total of 73 primary tumors, with a median of 8 primary tumors per patient (range, 2–14). Each primary tumor had a median of 1,194 (range, 350–2,757; IQR, 991–1,493) SNVs and indels. In six of the 11 patients, 2–14 variants were shared across multiple primary tumors, whereas in the remaining five patients, primary tumors had zero or one shared variant with others. For example, in patient #876, four out of eight primary tumors (P2, P3, P7, and P8) shared 10 variants (chr11:13073563C>G, chr12:60790192C>A, chr14:65344107C>T, chr21:21695571G>A, chr22:27162797T>C, chr4:107552573C>T, chr5:88906833G>A, chr6:145946753T>A, chr8:50544452C>A, and chr9:127337624C>T). Specifically, primary tumors P2 and P3 had 1,027 and 1,484 variants, respectively, and the two primary tumors shared 12 variants. The probability of such an overlap occurring by chance, based on a hypergeometric model, is 3.5e^−49^, assuming there are up to 3 billion possible mutation nucleotides in the genome and that each SNV occurs independently. Therefore, such an overlap is extremely unlikely to occur by chance alone.

**Figure 6:**
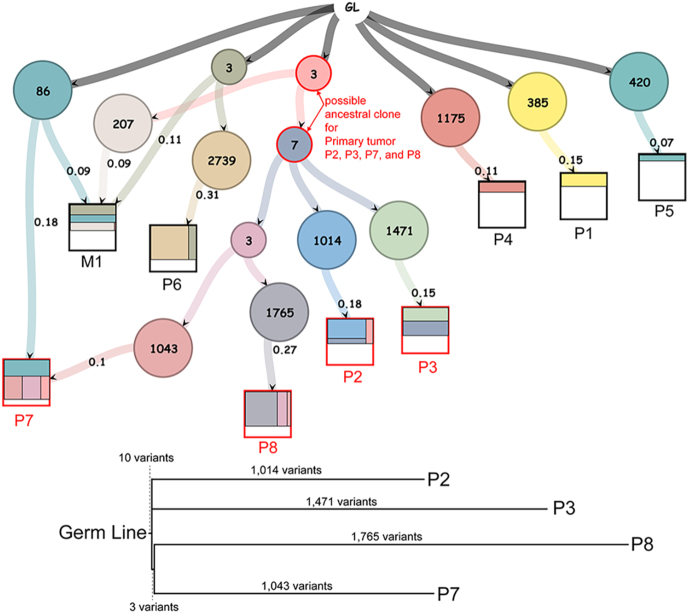
Additional genomic subclonal composition analysis. (Top) Subclonal compositions and a lineage tree of multiple SI-NET tumors in patient #876 were inferred based on variant allele frequencies using an open-source dataset (24). Each circle represents a clone, with the number inside indicating the number of acquired variants. Arrows indicate the direction of tumor evolution. Squares represent primary tumors (P1–P8) or lymphatic metastasis (M1), with colors inside the squares reflecting the relative proportions of different clones. The number of arrows connecting clones to tumors corresponds to the clonal composition percentage. GL, germline variants. (Bottom) A maximum parsimony phylogenetic tree illustrates the potential ancestral relationship among tumors P2, P3, P7, and P8, with branch lengths indicating the number of variants as scaled.

The LICHeE program inferred components of cell subpopulations in each tumor based on variant allele frequencies (VAFs). The different tumors were composed of varying cell subpopulations with distinct clonalities, and in patient #876, P2, P3, P7, and P8 shared a possible common ancestral clone in their limited cell subpopulation (VAFs: 18, 15, 10, and 27%, Fig. 6).

Similar patterns of possible common ancestral clones were identified in the other five patients: among two tumors in patient #772; five tumors in patient #848; nine tumors in patient #852; five tumors in patient #947; and four tumors in patient #952 (Supplementary Fig. 1). In total, 28 out of 73 primary tumors across the cohort exhibited such clonal overlap in their limited subpopulations (VAFs – median: 17%, range: 8–56; IQR: 14%, 25%).

## Discussion

In this study, we identified and characterized subclinical, microscopic neuroendocrine tumor foci in the superficial layers of the small intestine, which we termed small intestinal neuroendocrine tumorlets (SINTs). SINTs were identified both in multifocal and unifocal SI-NET (50 and 30%), and comparative analysis found that ‘multifocal-spectrum’ (defined as multifocal or unifocal with SINT) SI-NET cases were associated with higher BMI and local aggressiveness compared with ‘true unifocal’ cases (unifocal without SINT).

Prior studies have shown that survival and clinicopathological characteristics of multifocal and unifocal SI-NET are similar (6, 7, 27). In the current study, prognostic factors such as distant metastasis or PFS were not clearly different between ‘multifocal-spectrum’ and ‘true unifocal’ SI-NETs; however, we found that ‘multifocal spectrum’ was associated with higher pT stage, higher frequency of PNI, and larger mesenteric mass. Moreover, the disease duration time between the two groups, a possible confounder, is likely comparable with their similar tumor sizes, a primary indicator for lead-time bias, and their similar tumor grades, which reflect the proliferation rate (28). Therefore, the pathological identification of SINT might serve as an indicator of ‘multifocal spectrum’ and local aggressiveness, which would have been overlooked in previous studies relying on clinical multi- or unifocality.

Many of the SINTs were located in separate intestinal folds, with a median distance of 2 mm from the nearest mass, consistent with the typical thickness of the intestinal fold (1.8–2.1 mm) (29). These SINTs can include two different entities: newly developing small lesions, because enterochromaffin cells in the superficial layer are the probable origin of SI-NETs (30, 31), and disseminated tumor foci, because several SINTs were observed within lymphatic vessels, consistent with tumor spread via superficial lymphatics. Typically, intestinal lymphatic flow, along with dietary lipids absorbed by the intestinal epithelium, is directed vertically to the mesentery and ultimately reaches the thoracic duct (32). However, mucosal lymphatic ducts have lateral communications that allow lateral distribution of lymphatic fluid and immune cells along the intestine (33, 34, 35). Moreover, obesity causes mucosal lymphatic remodeling with dilation or disorganization, which aligns with the observed association between higher BMI and SINTs (36, 37). Furthermore, this lateral pathway can be enhanced when the vertical flow is obstructed, and the small intestine of SI-NET patients with mesenteric metastasis demonstrated longitudinal lymphatic drainage >18 cm by blue dye injection (38, 39). These reports might explain the larger mesenteric mass in multifocal cases. The previously reported asymmetric distribution of multifocal SI-NETs, predominantly on the left side of the major arterial axis, may be explained by lateral tumor cell spread and vertical lymphatic drainage along the major vessels (40). Of note, out of 11 patients without lymphovascular invasion reported in the original pathology assessment, SINTs were identified in four patients (36%). This suggests that SINTs may be under-recognized during routine lymphovascular invasion evaluation.

Then, we hypothesized that these disseminated tumor cells might give rise to new primary tumors. Our genomic re-analysis detected shared variants in a limited cell subpopulation (VAFs: 17%) of 38% (28/73) of the multiple tumors. This suggests that approximately 13% (38% × (17%/50%)) of the cell population in multifocal SI-NET primary tumors might share a clonal ancestral origin with other primary tumors within the same patient.

There might be two alternative explanations for the shared variants. One is that disseminated tumor foci might have merged into a pre-existing tumor mass, as suggested in P7 in Fig. 6, and did not contribute to its development. Here, the number of the shared variants was relatively low (2–14) compared to the total number of variants that each tumor harbored (median 1,194). This suggests that the common ancestral truncal clone likely had diverged at a very early stage in the development of the parent tumor and that significant time had passed since they merged into another tumor, rather than recently diverged cells merging into an older pre-existing tumor mass. The other possible explanation is that the shared variants developed independently by specific selection pressures, functioning similarly to driver mutations. However, no common shared variants were observed across patients (Supplementary Table 2). Therefore, these alternative explanations are considered unlikely, and it is reasonable to conclude that clonal spread via disseminated tumor foci, a part of SINTs, might play a role in the development of subpopulations across multiple tumors.

Two other studies have investigated the clonality of multifocal SI-NETs (25, 41). Elias *et al.* reported 1–8 shared variants among subsets of tumor pairs, concluding that these were too few to indicate clonality (25). However, the probability of two shared variants out of a total of 1,200 variants by chance is still only 1.1e^−7^, suggesting that such an overlap is unlikely to be incidental. They proposed an alternative explanation with early developmental lineage-specific mutations. Notably, the dataset re-analyzed in this study had used adjacent normal mucosa as a control, which clarified that the shared ancestral clones were still distinct from the adjacent normal intestine. Patte *et al.* found a higher range of shared mutations (8–89), possibly due to differences in mutation filtering criteria or inclusion of various tumor regions in sampling process, given the subclonal heterogeneity of the tumors (41).

This study has some limitations. First, this study is exploratory and hypothesis-generating with a relatively small number of patients because this is the first study to characterize and define subclinical microscopic lesions in primary SI-NETs. The analysis detected differences with large effect sizes (BMI, 0.67; PNI, 0.73; mesenteric mass size, 0.68; and T stage, 0.88) but was underpowered for detecting small to medium differences, particularly, in the SINTs’ prevalence by clinical multifocality (effect size 0.41), and in distant metastasis by ‘multifocal spectrum’ (effect size 0.53). For the observed OR of 2.3 (SINTs’ prevalence) and 3.1 (distant metastasis) to reach statistical significance, sample sizes of 186 and 113 patients would be required, respectively. Further validation studies need such sample sizes in order to confirm these small to medium differences.

Second, the progression of low-grade SI-NET is slow, and our median follow-up of 42 months may be insufficient for analyzing overall survival. Given this limitation, we focused on PFS and observed 15 progressions among 40 patients, a potentially meaningful proportion for the low-grade SI-NET population. For the observed HR of 1.4 to reach statistical significance, a sample size of 914 patients would be required, but its clinical significance may still be modest with the low HR in the baseline low-risk disease.

Third, the histopathological review was performed with archived slides from representative sections, rather than the whole-sections of the resected intestines, which may have underestimated the prevalence of SINTs. While whole-section preparation would be ideal, the substantial cost and labor requirements made this impractical for this pilot study. Moreover, the SINT adjacent to a palpable mass could represent an extension of the palpable mass rather than a separate focus. In either case, the presence of SINT may indicate a greater lateral expansiveness in the superficial layers of SI-NETs with SINT compared to those without, extending beyond the hypothetical circumference of the palpable mass to reach the adjacent separate fold of the small intestine.

Finally, our interpretation of genomic re-analysis needs appropriate caution, because this is based on several assumptions, such as the mutation was heterozygous and there was no copy number alteration; all of the shared clones originate from disseminated tumor foci; and all the cell subpopulations were captured properly in the sampled tissues.

## Conclusion

SINTs may serve as a new biomarker for ‘multifocal spectrum’ with local aggressiveness. The actual frequency of multifocal SI-NET may be higher than currently recognized in clinical practice. Further studies are needed to validate their prognostic significance and biological role and to make clinical recommendations.

## Supplementary materials







## Declaration of interest

The authors declare that there is no conflict of interest that could be perceived as prejudicing the impartiality of the work reported.

## Funding

This work was supported by The Neuroendocrine Tumor Research Foundation, The Placzek Family Foundation, and Larry and Margaret Hauben.

## Author contribution statement

AY, NA, GEK, and EKN contributed to conceptualization; AY, GEK, and EKN helped in methodology; AY, AP, LZ, and PL contributed to formal analysis; AY, GEK, and EKN helped in investigation; AY wrote the original draft; AY, NA, GEK, AP, LZ, PL, EKB, and EKN helped in writing – review and editing; EKN contributed to funding acquisition; GEK, KH, CUC, and, EKN helped in resources; and EKB and EKN supervised the study. All authors read and approved the final manuscript.
