# Supplementary material for: Characterization of small intestinal neuroendocrine tumorlets
Source: Endocr Relat Cancer. 2026 Apr 13;33(4):e250425. doi: 10.1530/ERC-25-0425 (PMC13097121; doi:10.1530/ERC-25-0425)
Supplement: Supplementary file 1 [file supplementary_figure.pdf]

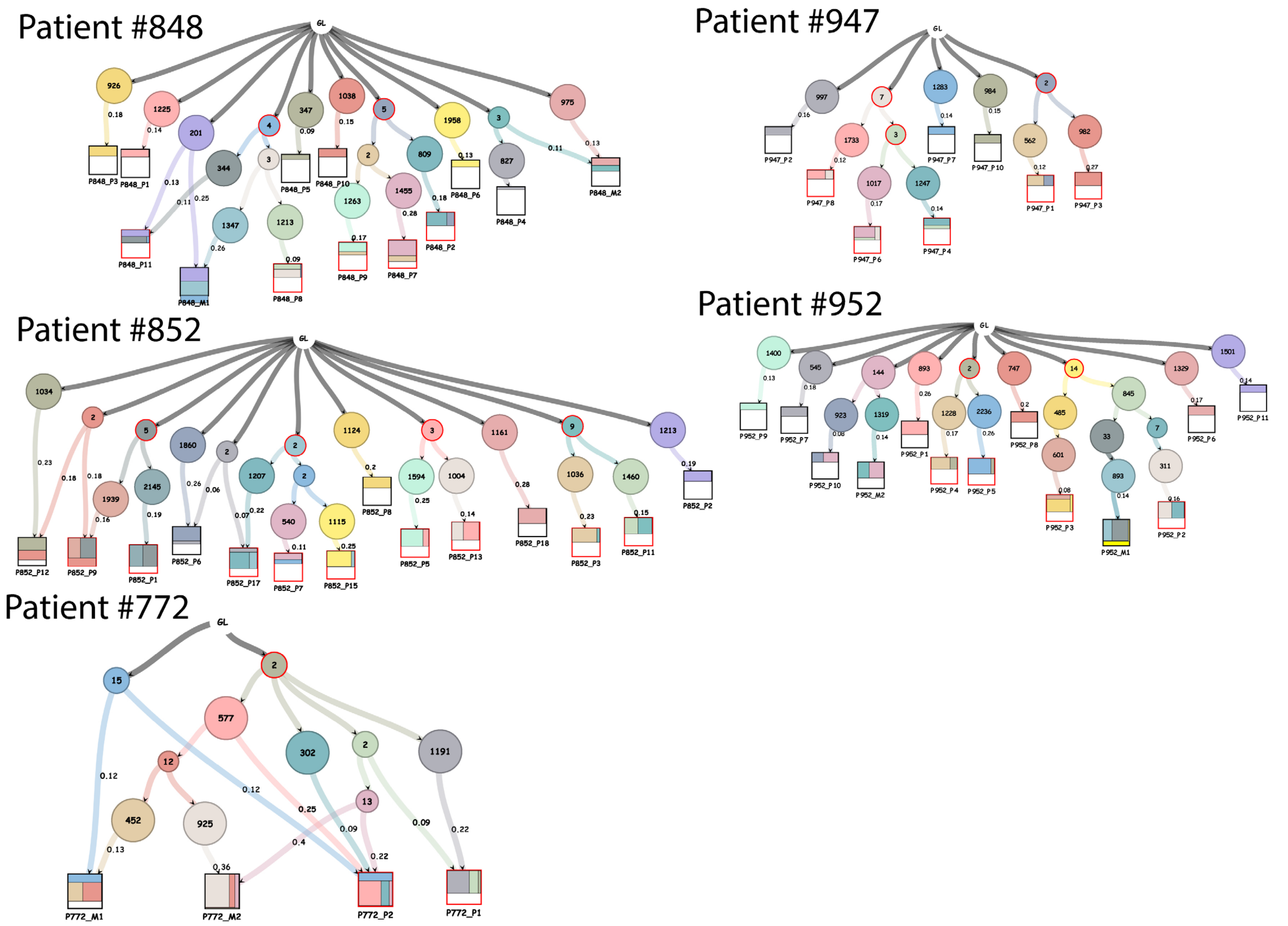

Supplementary Fig. 1 Additional genomic subclonal composition analysis of the other five patients. Subclonal compositions and a lineage tree of multiple SI-NET tumors in Patient #848, #852, #947, #952, and #772 were inferred based on variant allele frequencies using an open-source dataset (24). Each circle represents a clone, with the number inside indicating the number of acquired variants. Arrows indicate the direction of tumor evolution. Squares represent primary tumors (P1–P18) or lymphatic metastasis (M1-2), with colors inside the squares reflecting the relative proportions of different clones. The number of arrows connecting clones to tumors corresponds to the clonal composition percentage. GL, germline variants.
