# Supplementary material for: Characterization of small intestinal neuroendocrine tumorlets
Source: Endocr Relat Cancer. 2026 Apr 13;33(4):e250425. doi: 10.1530/ERC-25-0425 (PMC13097121; doi:10.1530/ERC-25-0425)
Supplement: Supplementary file 2 [file supplementary_table_1.pdf]

Supplementary Table 1 Demographics of the cohort by clinical multifocality.

Characteristic	N	multifocality, N (%)		p-value
		Uni-, N = 20	Multi-, N = 20	
Age, Median (IQR)	40	64 (51, 69)	64 (52, 68)	0.89
Gender Female	40	10 (50)	6 (30)	0.33
BMI (kg/m2), Median (IQR)	40	25.6 (22.0, 31)	27.4 (24.9, 30)	0.22
Race White	40	15 (75)	14 (70)	1
Carcinoid syndrome	40	8 (40)	8 (40)	1
Urine 5-HIAA (mg/24h)	25	33 (6, 83)	10 (7, 26)	0.40
History of small bowel obstruction	40	4 (20)	5 (25)	1
Size of the largest primary tumor (cm), Median (IQR)	40	2.0 (1.2, 3.0)	1.9 (1.3, 3.0)	0.80
Tumor Grade	40			0.50
1		12 (60)	14 (70)	
2		8 (40)	5 (25)	
3		0 (0)	1 (5.0)	
Number of primary tumors, Median (IQR)	40	1 (1, 1)	4 (2.8, 9)	$7.5e^{-9}$
Perineural invasion	40	13 (65)	18 (90)	0.12
Lymphovascular invasion	40	15 (75)	14 (70)	1
Size of mesenteric mass (cm), Median (IQR)	40	1.6 (0.0, 2.8)	3.0 (1.8, 3.9)	**0.045**
T Staging	40			0.064
T1/T2		8 (40)	2 (10)	
T3/T4		12 (60)	18 (90)	
N +	40	17 (89)	20 (100)	0.23
M Staging	40			1
M0		13 (65)	12 (60)	
M1a/1b/1c		7 (35)	8 (40)	
Stage	40			0.58
I		2 (10)	0 (0)	
III		11 (55)	12 (60)	
IV		7 (35)	8 (40)	
Positive local surgical margin	40	0 (0)	1 (5.0)	1
Postoperative complications >= G3	40	0 (0)	0 (0)	NA
Survival status Dead	40	1 (5.0)	1 (5.0)	1
Follow-up months, Median (IQR)	40	42 (34, 59)	41 (33, 54)	0.82

BMI, Body mass index; 5-HIAA, 5-hydroxyindoleacetic acid; NA, not applicable
